# Mechanism of Action of Recombinant Acc-Royalisin from Royal Jelly of Asian Honeybee against Gram-Positive Bacteria

**DOI:** 10.1371/journal.pone.0047194

**Published:** 2012-10-09

**Authors:** Lirong Shen, Dandan Liu, Meilu Li, Feng Jin, Meihui Din, Laurence D. Parnell, Chao-Qiang Lai

**Affiliations:** 1 Department of Food Science and Nutrition, School of Biosystems Engineering and Food Science, Zijinggang Campus, Zhejiang University, Hangzhou, Zhejiang, China; 2 Nutrition and Genomics Laboratory, Jean Mayer-USDA Human Nutrition Research Center on Aging at Tufts University, Boston, Massachusetts, United States of America; Nanyang Technological University, Singapore

## Abstract

The antibacterial activity of royalisin, an antimicrobial peptide from the royal jelly produced by honeybees, has been addressed extensively. However, its mechanism of action remains unclear. In this study, a recombinant royalisin, RAcc-royalisin from the royal jelly of Asian honeybee *Apis cerana cerana*, was expressed by fusing with glutathione S-transferase (GST) in *Escherichia coli* BL21, isolated and purified. The agar dilution assays with inhibition zone showed that RAcc-royalisin, similar to nisin, inhibits the growth of Gram-positive bacteria. The antibacterial activity of RAcc-royalisin was associated with its concentration, and was weakened by heat treatment ranging from 55°C to 85°C for 15 min. Both RAcc-royalisin and nisin exhibited the minimum inhibitory concentrations (MIC) of 62.5 µg/ml, 125 µg/ml, and 250 µg/ml against Gram-positive bacterial strains, *Bacillus subtilis* and *Micrococcus flavus* and *Staphyloccocus aureus* in the microplate assay, respectively. However, RAcc-royalisin did not show antimicrobial activity against tested Gram-negative bacterial and fungal strains. The antibacterial activity of RAcc-royalisin agrees well with the decrease in bacterial cell hydrophobicity, the leakage of 260-nm absorbing materials, and the observation by transmission electron microscopy, all indicating that RAcc-royalisin induced the disruption and dysfunction of cell walls and membranes. This is the first report detailing the antibacterial mechanism of royalisin against Gram-positive bacteria, and provides insight into the application of recombinant royalisin in food and pharmaceutical industries as an antimicrobial agent.

## Introduction

Royalisin, a potent antimicrobial peptide (AMP), was first isolated from royal jelly of the Western honeybee *Apis mellifera* [1]. The mature royalisin peptide from the Western honeybee consists of 51 amino acid residues, with a molecular weight of 5.52 kDa, belonging to the family of defensins, the most common group of AMPs in insects [2], [3]. Defensins are small, *β-*sheet-rich, cationic peptides, and have amphiphilic properties, which are central for their antimicrobial activities [4]. All defensins with distinct structures have been categorized into three subfamilies: classical defensins, *β*-defensins and insect defensins. Classical defensins and *β*-defensins that are found in mammalian neutrophils and play important roles in defending against Gram-positive and Gram-negative bacteria, mycobacteria, fungi, and some enveloped viruses. Insect defensins act against Gram-positive bacteria, but Gram-negative bacteria are generally resistant to their action. Protein sequences of insect defensins share only limited sequence similarity with mammalian defensins, but do share a common structure comprising an amino-terminal loop, R-helix and two anti-parallel *β*-strands stabilized by disulfide bridges [5].

Royal jelly (RJ) royalisin of the Western honeybee *Apis mellifera* is one of the main active constituents which combat Gram-positive bacteria and fungi [1], [6], [7]. Although with a narrower antibacterial spectrum than native RJ, royalisin strongly inhibits the growth of Gram-positive bacteria including *Clostridium, Corynebacterium, Leuconostoc, Staphylococcus*, and *Streptococcus* at the effective concentration of 1 µM, the antibacterial potency of which is comparable to that of native RJ at 10 µg/ml. Royalisin also inhibits other Gram-positive bacteria, such as *Bacillus subtilis, Sarcina lutea*, American foulbrood caused by a Gram-positive bacterial pathogen, Paenibacillus larvae larvae [7], [8], The recombinant royalisin fused with oleosin central hydrophobic domain, and reconstituted with triacylglycerol and phospholipids to form artificial oil bodies could inhibit several Gram-negative bacteria, including *Pseudomonas aeruginosa, Salmonella choleraesuis* and *Vibrio parahaemolyticus* [9]. Moreover, the royal jelly peptide fraction also has been demonstrated to have antifungal activity inhibiting the model fungus *Botrytis cinerea* [7], a plant pathogen, and *Alternaria brassica.* However, no inhibitory activity against *E. coli* was observed [9]. Therefore, it has been well known that royalisin is valuable both for the prevention of honeybee diseases and RJ preservation. Due to its safety and effectiveness, royalisin is a potential AMP for food preservation, therapeutic application and medication.

The Asian honeybee, *Apis cerana cerana* has been domesticated and maintained by farmers for over 3000 years in China. It was recently reported that many more hymenoptaecin peptides are found in Asian honeybee than those in the Western honeybee (13 versus 1) [10]. In total, 29 different defensin cDNA sequences encoding seven different defensin peptides were amplified and identified in the honeybee hemocytes. Nearly two thirds (20/29) of the retrieved cDNAs coded for the major peptide, royalisin, and one or two cDNAs coded for other defensin peptides. This analysis also revealed that the rich royalisin in the hemocytes is associated with the stronger tolerance and resistance toward the mite *Varroa destructor* and the microsporidan *Nosema ceranae*, as well as other pathogens toward the Asian honeybee than those in the Western honeybee [10].

In previous work [11], we cloned and expressed the Asian honeybee Acc-royalisin gene to demonstrate unequivocally that Acc-royalisin possesses antibacterial activity against Gram-positive bacterial strains, *B. subtilis* and *Micrococcus flavus* as well as *Staphyloccocus cerevisiae*. This suggested that RAcc-royalisin refolded to its functional structure and its biological activity could be assayed. In this work, we extend those findings to offer clarifying details on the properties, minimum inhibitory concentration (MIC), and mechanism of anti-Gram-positive bacterial action.

## Results

### Expression and Purification of RAcc-royalisin

**Figure 1:**
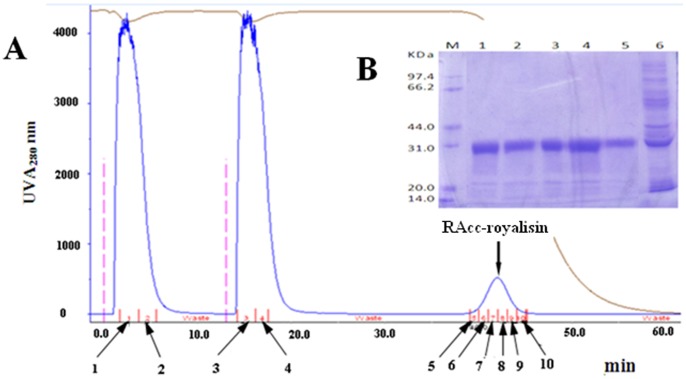
Purification diagram and SDS analysis of RAcc-royalisin. (A) The diagram of RAcc-royalisin purified at UV A_280_ nm from the recombinant *E. coli.* 1–2 (indicated by arrows): The outflow peak of the first injection samples; 3–4 : The outflow peak of the second injection samples; 5–10 : The elution peak of RAcc-royalisin. (B) The SDS-PAGE pattern of the purified RAcc-royalisin. Lane M: Protein marker; Lane 1–5: The elution peak products; Lane 6: The outflow peak products.

To obtain a large quantity of RAcc-roylisin, we have developed a new method to express and purify proteins using GSTrap FF column connected to GE ÄKTA Explorer 100 FPLC-Fast Protein Liquid Chromatography System. The column chromatography showed one of the elution peaks as the RAcc-royalisin (Figure 1A). The SDS-PAGE gel profile of the elution peak sample indicated the protein band with estimated molecular weight of 32 kDa (Figure 1B)was identical to RAcc-royalisin we described previously [11]. RAcc-royalisin was composed of GST (26 kDa) and Acc-royalisin (5.52 kDa) containing 51 amino acid residues [11]. Using the Bradford method, we estimated that the yield of purified soluble RAcc-royalisin was 1.5 mg/l of cell culture.

### Antibacterial Activity of RAcc-royalisin

**Figure 2:**
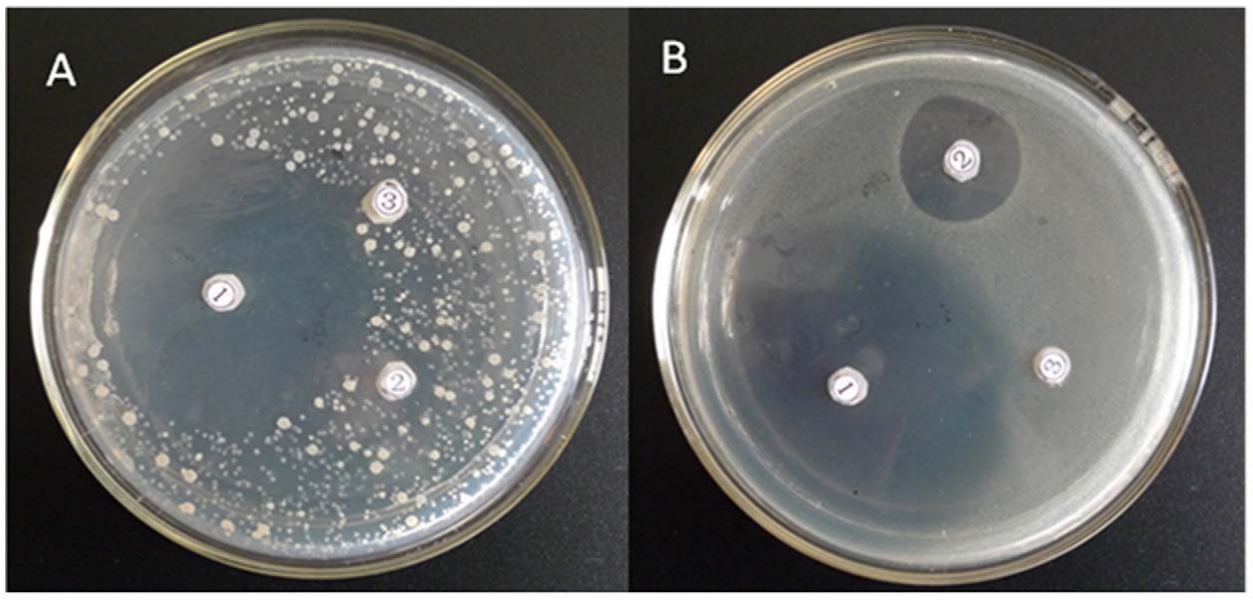
RAcc-royalisin inhibition plate against Gram-positive bacteria. *B. subtilis* (plate A) and *M. flavus* (plate B) were treated by RAcc-royalisin 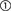, nisin 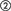 and blank control 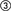, respectively.

The lyophilized soluble RAcc-royalisin diluted in sterilized ddH_2_O was used to assay inhibitory activity against Gram-positive bacterial strains, *B. subtilis* and *M. luteus*. We observed that both bacterial strains were inhibited by RAcc-royalisin, revealing an obvious inhibitory zone (Figure 2, position 1 for plates A, B) similar to that of nisin, a Gram-positive antimicrobial agent against the same bacterial strains (Figure 2, position 2 for plates A, B). No inhibition effect was observed for the blank control (Figure 2, position 3 for plates A, B).

**Figure 3:**
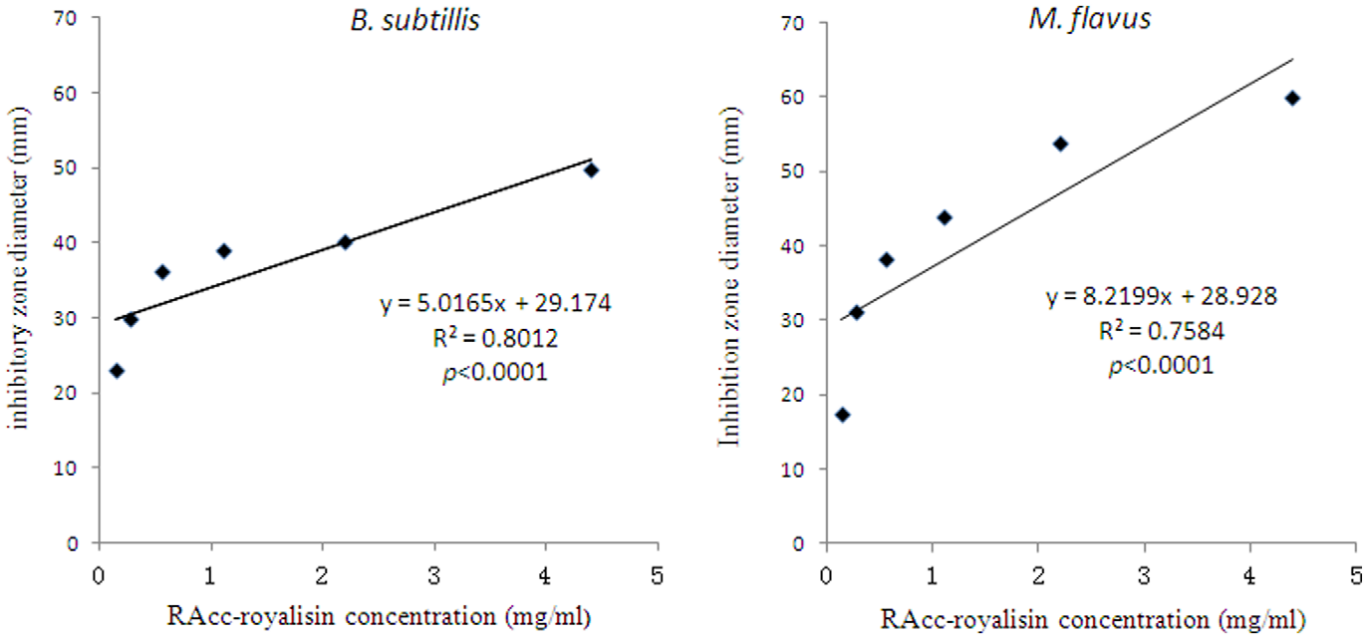
Correlation between RAcc-royalisin concentration and antibacterial activity against Gram-positive bacterial strains. Correlation between RAcc-royalisin concentration and antibacterial activity against *B. subtilis* (A) and *M. flavus* (B).

The relationships between the RAcc-royalisin concentration and growth inhibitory effects against *B. subtilis* and *M. flavus* are given in Figure 3. The concentration of RAcc-royalisin ranging from 0.138 to 4.400 mg/ml strongly and positively correlated with its antibacterial activity (*p*<0.0001). RAcc-royalisin at a low concentration of 0.138 mg/ml inhibited growth of *B. subtilis* and *M. flavus* effectively, whereby the inhibition zone diameters were 23.22±1.34 mm and 17.56±2.16 mm, respectively.

### MIC and Antimicrobial Spectrum of RAcc-royalisin

MIC is defined as the lowest concentration of an antimicrobial that will inhibit the visible growth of a microorganism after overnight incubation, which is the most often used to determine the *in vitro* activity of new antimicrobials [12]. The MICs for RAcc-royalisin and nisin against three Gram-positive bacterial strains, *B. subtilis*, *M. flavus* and *S. cerevisiae* are given in Table 1. The concentrations of both RAcc-royalisin and nisin ranging from 3.9 to 1000 µg/ml were significantly associated with its antibacterial activity (*p*<0.0001). The MICs for both RAcc-royalisin were 62.5 µg/ml against *B. subtilis,* 125 µg/ml *M. flavus*, and 250 µg/ml against *S. cerevisiae,* respectively (Table 1). In addition, RAcc-royalisin showed antibacterial activity against another Gram-positive bacteria, *Clostridium tetani* (MIC = 250 µg/ml, Table 2).

**Table 1:** The Δ*OD*_600_ (mean ± SD) value of RAcc-royalisn and nisin against three Gram-positive bacterial strains at different concentrations.

Strains	Concentration	1000	500	250	125	62.5	31.25	15.6	7.8	3.9	CK
	(µg/ml)										
	RAcc-Royalisin	0.0010	0.0075	0.0203	0.0304	0.3205	0.3167	0.3211	0.4167	0.4474	0.5764
*M. flavus*		±0.0006	±0.0022	±0.0026	±0.0006*	±0.0057	±0.0054	±0.0035	±0.0044	±0.0022	±0.0081
	Nisin	0.0103	0.0018	0.0013	0.0036	0.2832	0.3150	0.3167	0.3208	0.4160*	0.5764
		±0.0005	±0.0006	±0.0004	±0.0008*	±0.0055	±0.0008	±0.0054	±0.0039	±0.0050	±0.0081
	RAcc-Royalisin	0.0010	0.0011	0.0032	0.2822	0.3065	0.3178	0.3348	0.4068	0.4390	0.5956
*S. aureus*		±0.0002	±0.0003	±0.0004*	±0.0097	±0.0116	±0.0055	±0.0183	±0.0038	±0.0097	±0.0083
	Nisin	0.0258	0.0367	0.0396	0.1281	0.2707	0.2844	0.2931	0.3074	0.3284	0.3508
		±0.0106	±0.0060	±0.0008*	±0.0088	±0.0152	±0.0089	±0.0066	±0.0116	±0.0096	±0.0219
	RAcc-Royalisin	0.0010	0.0012	0.0032	0.0148	0.0183	0.3145	0.3428	0.4123	0.4430	0.6006
*B.subtilis*		±0.0001	±0.0002	±0.0004	±0.0045	±0.0018*	±0.0054	±0.0062	±0.0022	±0.0045	±0.0042
	Nisin	0.0071	0.0071	0.0135	0.0127	0.0058	0.1963	0.2144	0.2593	0.2643	0.2679
		±0.0043	±0.0053	±0.0042	±0.0099	±0.0018*	±0.0042	±0.0119	±0.0104	±0.0089	±0.0227

n = 3;

“*”:MICs are concentrations where the average Δ*OD*_260_ value was not greater than 0.05.

**Table 2:** Minimum inhibition concentrations (µg/ml; MIC^a^) at 24 h for RAcc-royalisin and nisin on different microorganism strains.

Microorganisms strains	RAcc-royalisin	Nisin
**Gram-positive bacteria**		
*B. subtilis*	<62.5	<62.5
*M. flavus*	<125	<125
*S. aureus*	<250	<250
*C. tetani*	<250	ND^b^
**Gram-negative bacteria**		
*E. coli*	>2000	ND
*S. typhimurium*	>2000	ND
*P. vulgaris*	>2000	ND
**Fungus**		
*A. oryzae*	>2000	ND
*P. viridicatum*	>2000	ND
*P. fermentans*	>2000	ND

a MICs are concentrations where the average Δ*OD*_600_ value was not greater than 0.05.

b ND: not detected.

Meanwhile, we investigated the antimicrobial spectrum of RAcc-royalisin through measuring its MICs against other six microorganism strains (Table 2). However, RAcc-royalisin did not showed antimicrobial activity for three tested Gram-negative bacterial strains, *E. coli, Salmonella typhimurium and Proteus vulgaris,* and three tested fungal strains, *Aspergillus oryzae, Penicillium viridicatum* and *Pichia pastoris* (MIC>2000 µg/ml).

### Thermostability Assay of RAcc-royalisin

**Figure 4:**
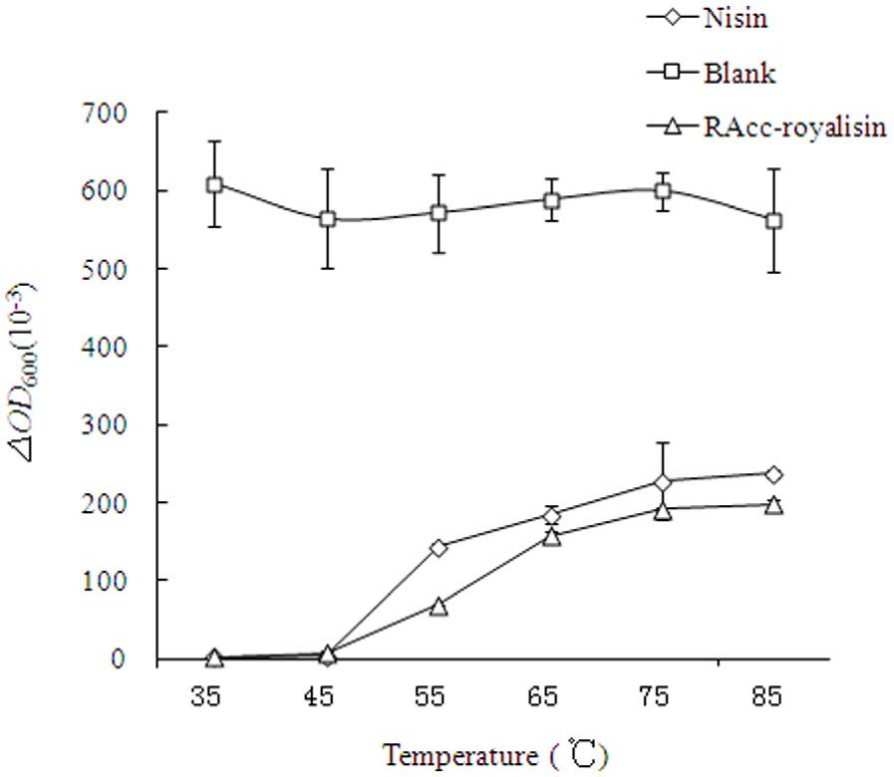
The effects of temperature on the antibacterial activity of RAcc-royalisin against *B. subtilis*.

The antibacterial activity of heat-treated RAcc-royalisin and nisin (1000 µg/ml) at different temperatures ranging from 35 to 85°C against *B. subtilis* was assessed as shown in Figure 4, providing important measures of the thermolabile nature of royalisin. After 15-min treatments at 35, 45, 55, 65, 75 and 85°C, RAcc-royalisin and nisin exhibited similar antibacterial activity. The antibacterial activity (Δ*OD* value) of both RAcc-royalisin and nisin were negatively associated with temperature (*p*<0.0001). The Δ*OD* value for 37°C and 45°C was significantly greater than those for 55°C, 65°C, 75°C and 85°C, suggesting that the antibacterial activity of RAcc-royalisin is sensitive to temperatures higher than 45°C. However, both RAcc-royalisin and nisin still showed better antibacterial activity when the temperature was higher than 55°C compared to the blank control.

### Cell Surface Hydrophobicity and Membrane Permeability

**Figure 5:**
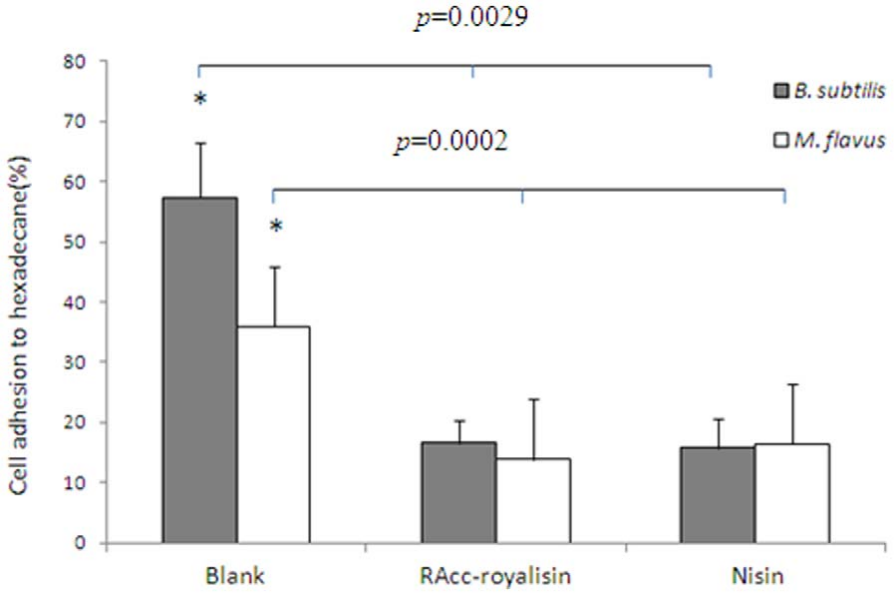
Determination of cell surface hydrophobicity as measured by cell adhesion.

The ability of RAcc-royalisin to alter cell surface hydrophobicity of *B. subtilis* and *M. flavus* was evaluated by the percentage of bacterial cells adhering to hexadecane (Figure 5). Compared with a blank control, RAcc-royalisin significantly decreased the bacterial cell surface hydrophobicity of *B. subtilis* from 65.8% to 19.7% (*p = *0.0029), and *M. flavus* from 39.7% to 19.6% (*P*-value* = *0.0001), respectively. There was no significant difference between RAcc-royalisin and nisin.

**Figure 6:**
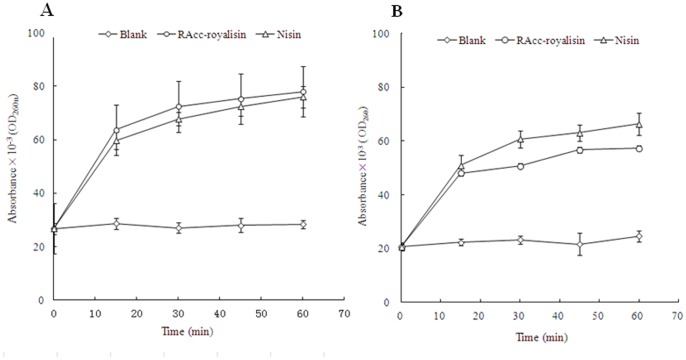
The effect of RAcc-royalisin on the cell membrane of Gram-positive bacteria. The UV absorbance of *B. subtilis* (A) and *M. flavus* (B) at 0, 15, 30, 45 and 60 minutes of treatment with RAcc-royalisin, nisin and blank control, respectively.

*B. subtilis* and *M. flavus* cell membrane permeability was evaluated by UV absorbance at 260 nm, as shown in Figure 6. RAcc-royalisin could induce the release of 260 nm absorbing material, which we interpret to be mostly DNA and RNA. The absorbance of *B. subtilis* and *M. flavus* treated by RAcc-royalisin increased quickly from 0.026 and 0.021 at 0 minutes to 0.064 and 0.048 at 15 minutes, and reached 0.078 and 0.057 at 60 minutes, respectively. The absorbance values of RAcc-royalisin-treated samples were significantly higher than those of the negative control and similar to that of the positive control nisin. This result suggests that RAcc-royalisin disrupted cell membrane permeability of the tested bacteria.

### Transmission Electron Microscopy

**Figure 7:**
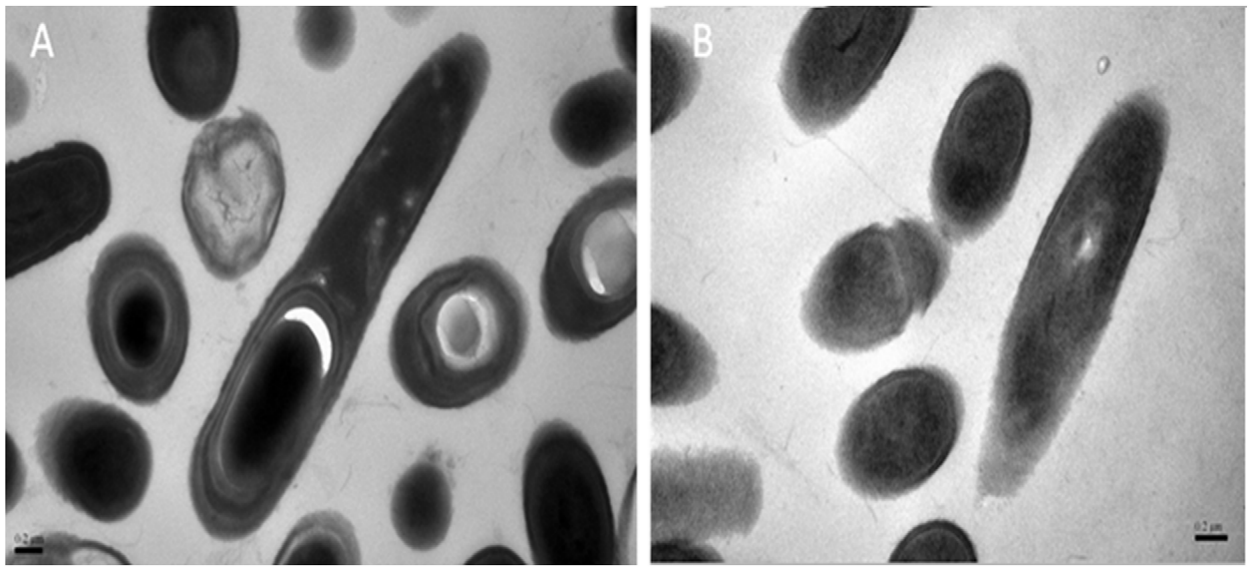
Transmission electron photomicrographs (at 50000× magnification) of *B. subtilis* treated with RAcc-royalisin. (A) Untreated *B. subtilis*. (B) *B. subtilis* treated with RAcc-royalisin at 37°C for 48 hours. Bar represents 0.2 µm.

The effect of RAcc-royalisin at MIC 50 µg/ml on the morphology of *B. subtilis* examined by transmission electron microscopy is shown in Figure 7. Untreated *B. subtilis* showed a typically structured nucleus and vacuoles. The cytoplasm contained several elements of endomembrane system and was enveloped by a typical cell wall structure (Figure 7A). When *B. subtilis* was treated with RAcc-royalisin at 50 µg/ml, however, greater damage was observed as noted by cell wall disruption, and consequently, cell death (Figure 7B).

## Discussion

If royalisin and other constituents of royal jelly from honeybees are to be used in food preservation, as many seek to do, basic characterization of these defensin peptides is necessary. Thus, in this study, RAcc-royalisin, expressed in *E. coli* and purified to a yield of 1.5 mg/l of cell culture, was shown to possess activity to inhibit the growth of Gram-positive bacteria similar to nisin, but did not showed antimicrobial activity for the tested Gram-negative bacteria and fungi strains. The antibacterial activity of RAcc-royalisin was associated with its concentration, and was decreased by heat treatment ranging from 55°C to 85°C. A concentration of RAcc-royalisin at 62.5 µg/ml inhibited growth of *B. subtilis*, and 125 µg/ml inhibited growth of *M. flavus*, 250 µg/ml was sufficient to combat growth of *S. aureus*, defining the MIC for these three species. The antibacterial activity of RAcc-royalisin at the MIC level was in good agreement with the decrease in bacterial cell hydrophobicity and the leakage of 260 nm absorbing material. Furthermore, the transmission electron microscope images indicated that RAcc-royalisin induced the disruption and dysfunction of the cell wall and membrane.

Antimicrobial peptides, or AMPs, functioning as novel antibiotics with minimal toxic and allergic side effects are of great interest due to their potential for therapeutic application, animal medication, and food preservation [13], [14], [15]. Because the production of AMPs from native sources in large quantities is at present not feasible, the *in vivo* biosynthesis of recombinant proteins is currently the most technologically reliable source of these polypeptides. Indeed, many AMPs have been obtained successfully through recombinant production in various heterologous hosts. Among the systems available for heterologous protein production, *E. coli* has been the most widely used host due to low cost, high productivity, and rapid acceptance for production of recombinant AMPs [9], [14]. However, difficulties have been encountered to obtain sufficient amounts of most recombinant AMPs from *E. coli* because of problems common to the expression of heterologous proteins, such as the formation of insoluble aggregates confined in inclusion bodies, the inability to post-translationally modify recombinant proteins [9], [16], susceptibility to cellular proteases, complicated purification from cell lysates, and toxic effects on host cells leading to low yields [14]. With the development of fusion expression systems, notably the *E. coli* GST-binding protein and *E. coli* 6×His, which produces correctly folded and soluble proteins of many heterologous genes, expression in bacterial cytoplasm has been successful. These systems may provide fused proteins with an opportunity to fold correctly, instead of precipitating into inclusion bodies although the reasons for this are unclear [17]. Based on this information, we were able to express a GST-Acc-royalisin fusion protein and purify the recombinant royalisin for the purpose of functional characterization. This offers a better strategy for effective production of AMPs.

Defensins are a major group of AMPs found widely in vertebrates, invertebrates and plants and exhibit bactericidal, fungicidal, anti-viral, and tumoricidal properties [9]. It was found that cell membranes are one target of these peptides, whereby disruption of that membrane leads to leakage of essential molecules resulting in cell death. Transmission electron micrographs of the Gram-positive bacteria *M. luteus* treated with tick defensin, a synthetic bactericidal, showed lysis of the cytoplasmic membrane and leakage of cellular cytoplasmic contents. These findings suggest that the primary mechanism of action of defensin is bacterial cytoplasmic membrane lysis [18]. The mode of action of a recombinant insect defensin from the blood of larvae of the flesh fly *Phormia terranova* against *M. luteus* has been addressed. It was shown that the defensin forms voltage-dependent channels in the bacteria, and disrupts the permeability barrier of the cytoplasmic membrane of the bacteria, resulting in loss of cytoplasmic potassium, a partial depolarization of the inner membrane, a decrease in cytoplasmic ATP, and inhibition of respiration [19]. The mechanism of action for defensin is similar to nisin, a widely used preservative against Gram-positive bacteria in food and dairy industries, which incorporates into the bacterial plasma membrane, rendering it permeable to ions [19], [20]. As one member of insect defensins, it has been known that royalisn shares common structural motifs with other insect defensins [11]. Although the mechanism by which royalisin functions was speculated according to other insect defensins [9], its actual mode of action remained unclear until now. Our work is the first to demonstrate that royalisin decreases bacterial cell hydrophobicity and disrupts cell membrane permeability in certain Gram-positive bacteria, and this induced the disruption and dysfunction of the bacterial cell walls and membranes.

Defensins are now considered promising candidates of new antibiotics among the AMPs. MIC values are related to and dependent upon both the defensin itself and the variety of bacterial species against which that defensin is tested. The MICs of the synthetic Ornithodoros defensin A against Gram-positive bacterial species, *B. cereus, B. subtilis* and *Staphyloccocus aureus* and *Enterococcus faecalis* were 20, 0.1, 1 and 0.75 µg/ml, respectively. The MICs of three defensins, Ornithodoros defensin A, Oryctes defensin and Mellitin against *M. luteus* were 0.1, 5.0 and 1.0, respectively [18]. Most insect defensins are active against a broad spectrum of bacteria and fungi at a concentration ranging from 1 to 100 µg/ml [9]. The royalisin isolated from royal jelly of the Western honeybee showed its bactericidal and fungicidal activity at a concentration of 5.4–180 µg/ml [7]. The MICs of recombinant royalisin against Gram-positive bacterial species ranged from 0.36 to 2.18 µg/ml [9]. The MIC against Gram-positive bacterial of RAcc-royalisin in our work ranged from 62.5 to 250 µg/ml. Our previous report has proved that the lower antibacterial activity of RAcc-royalisin was the result of the GST peptide in the fusion protein [11].

Our research in conjunction with previous reports has shown that royalisin possesses a broad antimicrobial spectrum, including all tested Gram-positive bacteria and many Gram-negative bacteria and fungi [9], [11]. Royalisin is considered stable at low pH and high temperature because of three disulfide bonds [1], [9]. Better stability has been found in other defensins, such as plectasin, which showed anti-*S. aureus* activity over a wide pH range of 2.0 and 10.0, a high thermal stability at 100°C for 1 hour and remarkable resistance to papain and pepsin [21]. Moreover, royalisin is a food-borne antibacterial peptide from royal jelly and possesses biological characteristics similar to nisin [11]. Hence, the royalisin peptide is certainly a potential target for its application to food preservation and human therapeutics. The successful expression and purification of high-yield RAcc-royalisin in this study has provided important fundamental knowledge for further studies on the application of recombinant royalisin in food and pharmaceutical industries as an antimicrobial agent.

## Materials and Methods

### Chemicals

All chemical products used were of the highest purity grade, were obtained commercially, and were used without further purification. Water was double-distilled (ddH_2_O) and sterilized. Nisin (1000 IU/mg), one of the bacteriocins and an anti-Gram-positive bacterial peptide produced by the bacterium *Lactococcus lactis* subsp. *Lactis*, was from Lanzhou Weiri Biological Engineering Corporation (Lanzhou, China). TakaRa protein markers were from TakaRa Biotechnology Co., Ltd. (Dalian, China). GSTTrapTM FF column was purchased from GE Healthcare, USA.

### Microorganism Strains

The Gram-positive bacterial strains *B. subtilis* CMCC63501, *M. flavus* CMCC28001, *S. aureus*CMCC26003 and *C. tetani* ATCC19406, the Gram-negative bacterial strains *E. coli* CGMCC1.1139, *S. typhimurium* CGMCC1.1190 and *P. vulgaris* CGMCC1.1527, and fungal strains *A. oryzae* CGMCC3.4383, *P. viridicatum* CGMCC3.3254 and *P. pastoris* CCREMSDMCC011108 were preserved at the Department of Food Science and Nutrition, Zhejiang University. *E. coli* strain BL21 (DE3) containing RAcc-royalisin expression vector, pGEM-4T-2-Acc-royalisin, was constructed by our laboratory [11]. All bacterial strains were reactivated by incubating in sterile Luria-Bertani medium (LB: 10 g Polypepton, 5 g yeast extract, 10 g NaCl, and deionized water to 1000 ml, pH 7.0) at 37°C for 12–15 hours. All fungal strains were reactivated by incubating in sterile Potato Dextrose Medium at 27°C for 12–15 hours.

### Preparation and Purification of RAcc-royalisin

The RAcc-royalisin expressed in *E. coli* BL21 was prepared according to the method of as described [11]. After lysis by brief ultrasonic pulses on ice, the supernatants containing soluble RAcc-royalisin were harvested, and purified by GSTrap FF column adapted to GE ÄKTA Explorer 100 (Amersham Pharmacia) FPLC-Fast Protein Liquid Chromatography System according to the purification methods in the technical manual. After purification, soluble protein was dialyzed to remove salts, lyophilized and then examined by SDS-PAGE. The concentration of RAcc-royalisin in each sample was determined by the method of Bradford with a SpectraMax M5 microplate reader (Multi-Detection Microplate Readers, America).

### Antimicrobial Activity Assay

The antibacterial activity of recombinant Acc-royalisin was determined with the agar dilution method as described [7] with modifications. Experiments were conducted in Petri plates, where 1 ml of bacterial culture solution (1.0×10^5^ cfu/ml) was spread after mixing with 9 Nutrition Agar medium (Hangzhou Microbiological Agents Co., Ltd., China). Six-mm diameter paper filters were applied to the agar surface, to which was added either 10 µL of 4.4 mg/ml RAcc-royalisin solution, 3 mg/ml nisin solution as positive control or sterile ddH_2_O as negative control, followed by incubation at 37°C for 16–20 hours. Plates exhibiting zones of growth inhibition were scored as showing antibacterial activity. Cultures sensitive to the growth inhibition properties of RAcc-royalisin were then tested at different concentrations (0.138, 0.275, 0.550, 1.100, 2.200 and 4.400 mg/ml) of RAcc-royalisin. Plates with inhibition zones were photographed against a black background to accurately measure the diameter of the inhibition zone. All experiments were performed in triplicate.

### Determination of the Minimum Inhibitory Concentration (MIC) and Antimicrobial Spectrum

The MIC value was determined according to the micro plate method as described [22], with modification. 100 µl microbial culture solutions containing 10^5^ cells/ml of all microbial strains were dispensed into the wells of a flat bottom 96-well microtiter plate. 100 µl RAcc-royalisin solution at various concentrations was added to each well using a double dilution method with an initial concentration of 1000–2000 µg/ml. Nisin solution and sterile deionized water were served as a positive control and a blank control, respectively. The 96-well microtiter plate for bacteria and fungi were incubated at 37°C and 27°C for 24 hours, respectively. The absorbance of each well was read at 0 and 24 hours on a Multiskan MK3 microplate spectroplate spectrophotometer (Thermo Labsystems, Finland) at 260 nm, which was recorded as optical density 1 (OD_1_) and optical density 2 (OD_2_), respectively. Each peptide concentration was tested in triplicate. The difference between OD_1_ and OD_2_ was expressed as Δ*OD*_600_ value = OD_2_–OD_1_. The MIC value at 24 h was defined as the lowest peptide concentration where the average Δ*OD*_600_ value was not greater than 0.05. MICs values were used to express the antimicrobial activity and to determine the antimicrobial spectrum.

### Effects of Temperature on RAcc-royalisin

Eppendorf tubes containing 1 ml of bacterial culture of *B. subtilis* (1.0×10^5^ cells/ml) and 1000 µg/ml solution of RAcc-royalisin or nisin (a positive control) were first heated at either 35°C, 45°C, 55°C, 65°C, 75°C or 85°C for 15 minutes, respectively. Then 100 µl bacterial culture solutions from each treated eppendorf tube was dispensed into the wells of a flat bottom 96-well microtiter plate, whereas wells filled with 100 µl sterile deionized water were served as a blank control. The 96-well microtiter plate was incubated at 37°C for 24 hours. Meanwhile, 100 µl bacterial culture solution from each unheated bacterial culture (in eppendorf tubes) as described above was dispensed into the wells of a flat bottom 96-well microtiter plate as well. The absorbance of each well was read at 0 (the unheated) and 24 hours (after incubation at 37°C) on a Multiskan MK3 microplate spectroplate spectrophotometer (Thermo Labsystems, Finland) at 260 nm. The thermal stability was measured by comparing the difference between OD_0_ and OD_24_ (Δ*OD*_600_ value) of each temperature. Each temperature was tested in triplicate.

### Cell Surface Hydrophobicity

Cell surface hydrophobicity was determined according to the microbial adhesion to solvents method as described [23] with modification. Bacteria (*B. subtilis* and *M. flavus*) were suspended in 0.1 M KNO_3_ (pH 6.2) containing approximately 10^8^ cells/ml. The absorbance (OD) of each bacterial suspension was measured at 400 nm and recorded as A_0_. Bacterial suspensions were mixed with RAcc-royalisin samples at MIC, and incubated at 37°C for 1 hour. Sterilized ddH_2_O and nisin with the same concentration as RAcc-royalisin were used as negative control (blank control) and positive control, respectively. 0.4 ml hexadecane was added to 2.4 ml of each bacterial suspension, and the mixture was vortexed for 2 minutes. To allow complete phase separation of the mixture, the aqueous phase was removed after 15 minutes and optical density (OD) at 400 nm was measured and recorded as A_1_. Cell surface hydrophobicity was calculated by (1–A_1_/A_0_) × 100%.

### Cell Membrane Permeability of RAcc-royalisin

Cell membrane permeability was carried out as described [24], with modification. Bacteria (*B. subtilis* and *M. flavus*) were suspended in sterile normal saline containing approximately 10^7^ cells/ml. These suspensions were mixed with RAcc-royalisin samples at MIC, and incubated at 37°C. Negative control was sterile normal saline and postive control was nisin at the identical concentration as RAcc-royalisin. Each specimen was taken at 0, 15, 30, 45 and 60 minutes, filtered through a 0.2 µm-pore-size filter, with its optical density (OD) then measured at 260 nm.

### Transmission Electron Microscopy (TEM)

Cells, either untreated or treated with RAcc-royalisin samples at MIC, were fixed with 2.5% glutaraldehyde in phosphate buffer (pH 7.0) at 4°C for ∼12 hours, washed three times in phosphate buffer (pH 7.0), then postfixed with 1% osmium tetroxide for 1 hour and washed three times in phosphate buffer. Specimens were dehydrated through a graded series of ethanol washes (50%, 70%, 80%, 90% and 95%) for ∼15 minutes at each wash, dehydrated with 100% ethanol for 20 minutes and treated with acetone for 20 minutes. This was followed by successive washes in an acetone-isoamyl acetate mixture (v/v = 1∶1) for 1 hour, at v/v = 1∶3 for 3 hours, and then pure isoamyl acetate for ∼12 hours at 70°C. Specimens were stained by citric acid lead solution and acetic acid double oxygen uranium 50% ethanol saturated solution for 15 minutes, respectively, and then observed in JEM-1230 transmission electron microscope.

### Statistical Analysis

Data analysis was performed using SPSS 16.0 statistical software (SPSS, Inc., Chicago, Illinois, USA). Linear regression was used to determine the relationship between the inhibition zone diameter and RAcc-royalisin concentration. In general, experiments were performed in triplicate, and values reported as mean ± standard deviation (SD). One-way ANOVA with Duncan’s post hoc tests was used to determine differences between each pair of treatment groups. *p-*values were two tailed, and a *p*-value <0.05 was considered as significant.
